# Assessing computational reproducibility in *Behavior Research Methods*

**DOI:** 10.3758/s13428-024-02501-5

**Published:** 2024-09-25

**Authors:** David A. Ellis, John Towse, Olivia Brown, Alicia Cork, Brittany I. Davidson, Sophie Devereux, Joanne Hinds, Matthew Ivory, Sophie Nightingale, Douglas A. Parry, Lukasz Piwek, Heather Shaw, Andrea S. Towse

**Affiliations:** 1 School of Management, University of Bath, Bath, UK; 2 Department of Psychology, Lancaster University, Lancaster, UK; 3 Department of Information Science, Stellenbosch University, Stellenbosch, South Africa

**Keywords:** Research policy, Open science, Reproducibility, Transparency, Meta-research

## Abstract

Psychological science has thrived thanks to new methods and innovative practices. Journals, including *Behavior Research Methods* (*BRM*), continue to support the dissemination and evaluation of research assets including data, software/hardware, statistical code, and databases of stimuli. However, such research assets rarely allow for computational reproducibility, meaning they are difficult to reuse. Therefore, in this preregistered report, we explore how *BRM*’s authors and *BRM* structures shape the landscape of functional research assets. Our broad research questions concern: (1) How quickly methods and analytical techniques reported in *BRM* can be used and developed further by other scientists; (2) Whether functionality has improved following changes to *BRM* journal policy in support of computational reproducibility; (3) Whether we can disentangle such policy changes from changes in reproducibility over time. We randomly sampled equal numbers of papers (*N* = 204) published in *BRM* before and after the implementation of policy changes. Pairs of researchers recorded how long it took to ensure assets (data, software/hardware, statistical code, and materials) were fully operational. They also coded the completeness and reusability of the assets. While improvements were observed in all measures, only changes to completeness were altered significantly following the policy changes (*d* = .37). The effects varied between different types of research assets, with data sets from surveys/experiments showing the largest improvements in completeness and reusability. Perhaps more importantly, changes to policy do appear to have improved the life span of research products by reducing natural decline. We conclude with a discussion of how, in the future, research and policy might better support computational reproducibility within and beyond psychological science.

**Supplementary Information:**

The online version contains supplementary material available at 10.3758/s13428-024-02501-5.

## Introduction

*Software that doesn’t run is kind of pointless*

Steve Furber CBE FRS FREng (RMC – The Cave, 2020)

Psychological science has benefited enormously from the proliferation of new methods and innovative practices. Historically, this often involved the development of physical apparatus. For example, Beatty and Kahneman designed a protocol to measure pupillary responses to mental effort and emotional arousal. Here, an analogue camera was automatically triggered to capture pupillary changes over time, measured from the captured images using a ruler (Kahneman, 2011). Today, with computer hardware and processing power readily available, psychological science continues to develop new laboratory methods at pace. Naturally, this involves the curation of other research assets that support data collection, processing, visualization, and analysis (AlNoamany & Borghi, 2018). For example, PsychoPy and Psychtoolbox have become standard tools for the presentation of stimuli and recording of responses in psychophysical and cognitive experiments (Lin et al., 2022; Peirce, 2007; Peirce et al., 2019). Similarly, once data has been collected, open-source software such as jamovi or the programming languages R or Python can support free and replicable statistical analysis (The jamovi project, 2024).

Contemporary technological developments, including advances in connectivity, have, in turn, led to a proliferation of other emerging research tools, such as software that can take advantage of cloud-based services. This is utilized in Gorilla Experiment Builder, which allows researchers to develop and deploy experiments online (Anwyl-Irvine et al., 2020). While a typical psychological experiment may involve measuring a person’s response time via keyboard presses and mouse movements (Baumeister et al., 2007), newer mobile technologies such as smartphones and wearable devices allow for ‘out-of-the-lab’ (i.e., in situ) measures of cognition and behavior (e.g., Kieslich et al., 2020). Thus, for example, Tomko et al. (2019) designed an electronic smart lighter that can accurately quantify cigarette smoking. Smartphones, in particular, contain a collection of sensors and logging routines that are capable of capturing ‘in-the-moment’ behaviors, thoughts, feelings, and emotions, as well as generating information about a participant’s online and offline environment (Davidson et al., 2022; Geyer et al., 2019; Keil et al., 2020; Miller, 2012; Stieger et al., 2018). Further, by combining advanced graphical abilities with gamification, various cognitive tasks have been validated in the assessment of working memory, attention, and decision-making (Paletta et al., 2014). Alongside smartphones, the increasingly widespread availability of virtual and augmented reality provides even more opportunities for ecologically valid research. For example, while the bystander effect (the inhibiting influence of the presence of others on a person's willingness to help someone in need) can be challenging to study ethically, virtual reality can generate a variety of more realistic environments and social scenarios for such study (Rovira et al., 2021).

When research assets (e.g., data, statistical code, materials, and software/hardware) are properly maintained, they can provide widespread support to the academic community in the development of robust understandings of replicable phenomena. A variety of journals, including *Behavior Research Methods* (*BRM*), *Psychological Methods*, and *Advances in Methods* *and* *Practices in Psychological Science*, support the dissemination and evaluation of these new methods and associated software. However, ensuring that research assets will function for a long time is rarely easy. One reason why commercial-grade software, for example, is so expensive is *because* it involves regular testing and maintenance over a prolonged period. By contrast, tools used for research purposes are often developed by individuals or groups who are frequently under pressure to move on to the next project. Research assets can also have numerous dependencies that are beyond the control of the research team or be in a repository that is temporary or unstable (e.g., accessed via an author’s personal website). As a result, many of the assets documented in journal publications may be at risk.

### Sharing data

The value of sharing data-related assets is well established across disciplines (Thayer, 2021; Trisovic et al., 2022). Beneficiaries include individuals (e.g., Colavizza et al., 2020; Piwowar et al., 2007; Piwowar & Vision, 2013), groups, and scientific progress (e.g., Munafò et al., 2017). However, a data access statement to the effect that “data will be provided upon reasonable request” is often just ‘smoke and mirrors’ because the distinction between a reasonable and an unreasonable request is unclear, always assuming such requests are even acknowledged. For example, Gabelica et al. (2022) analyzed all of the articles published during January 2019 by BioMed Central involving 333 open-access journals. Of the 1792 manuscripts in which data availability statements indicated authors’ willingness to share their data, only 122 (6.8%) provided that data on request.

In response to growing concerns about research quality, some psychology journals have developed policies that aim to improve the sharing of data and other research assets. Many of these policies extend from recommended practices for open research (e.g., Nosek et al., 2015). However, while a journal may ask authors to comply with these guidelines, the resulting landscape can be very different in practice. For example, in April 2019, following the introduction of the Open Science Badges incentive, *Psychological Science* published its first issue in which all of the research articles received the Open Data badge. Unfortunately, the attainment of a badge does not equate to value per se. Crüwell et al. (2023) observed that while 14 articles therein provided at least some data and six provided analysis code, only one article was rated as exactly reproducible, with three rated as essentially reproducible with minor deviations.^1^ Across multiple journals, the presence or absence of data rarely acts as a binary distinction when it comes to determining an article’s future value to other psychologists. When considered as a single asset in isolation (e.g., without analysis code or associated software), researchers will struggle to reuse data and successfully replicate the reported results: “even where open data can be identified, the majority of these lacked completeness and reusability” (Towse et al., 2021a, p. 1455).

### Beyond data: Assets to support computational reproducibility

While psychology research has tended to focus on the success of open research practices that support data sharing (Bauer, 2022; Grahe, 2021; Towse et al., 2021a; Wilson et al., 2014), in software engineering other important aspects of hardware and software resilience are more prominent (e.g., Venters et al., 2018). This can include multiple reliances that relate to software dependencies or specific pieces of hardware. The *Software Sustainability Institute*, by way of response, is a UK-based national facility for building better software.^2^ General science journals such as *PLOS* *ONE* also provide specific recommendations for sharing software. Laurinavichyute et al. (2022) observed that the strongest predictor of whether an attempt to reproduce a result was successful was the presence of the analysis code, which increased the probability of reproducing reported results by almost 40%.

In recent years, psychology has moved beyond being a discipline in which research papers largely generate bespoke data. As the contents of *BRM* confirm, psychologists are now creating methodological innovations such as analytical procedures (e.g., statistical algorithms that improve the signal-to-noise ratio of behavioral data, or that automate and optimize analytic procedures to make better use of secondary data) and even software products (e.g., that allow others to collect new forms of psychological data). As a community, psychologists also engage in methodological work that creates and validates research materials that others can use, such as creating word norms or scales (e.g., the Glasgow Face Matching Test (Burton et al., 2010)). As assets, such materials are equally important because, following creation and validation, they provide a common research language across a community. Without them, with everyone needing to develop materials from the ground up, there would be substantial redundancy and idiosyncrasy in research efforts as well as opaque differences between materials that would inevitably stymie reproducibility.

In practice, and as with data sharing, innovation within a single psychology publication can include a combination of assets. For example, Lynott et al. (2020) provide multidimensional measures of perceptual and action strength for 40,000 English words (materials and data), which also includes an interactive web application that provides a tool to allow for straightforward navigation of the corpus (software). While, as well as similarities, there are well-acknowledged differences between data and software assets (e.g., Lamprecht et al., 2020), it remains useful to establish some common metrics and principles that bring these assets together (see also Hasselbring et al., 2020a, b). That is, we can attempt to specify those signs of sustainability that are likely to be important to both. For example, the provision of documentation for any research asset (whether a readme file that acts as a data dictionary, comments in the analytical or source code for software to accompany the product implementation, and/or entity-relationship diagrams (ERDs) for databases) should ensure, at least in principle, that if the asset stops functioning, other users can investigate what the fault(s) might be. Any lack of transparency concerning software code that drives methods and analytical processes raises issues pertaining to replication and reusability that are analogous to incomplete data sharing (Turner et al., 2020). Ensuring that datasets, statistical code, materials, and software/hardware can all be used in the long term might be described collectively as ensuring we achieve computational reproducibility (Sawchuk & Khair, 2021; Stodden et al., 2018). To illustrate the challenge, Hardwicke et al. (2020) examined a sample of papers published in *Cognition* between 2014 and 2017 and concluded that while mandatory open data policies can increase the volume and quality of data shared, very few of the papers supported full computational reproducibility.

### Current study: Assessing computational reproducibility in *Behavior Research Methods*

Given this plurality of contributory research products, for which *BRM* papers offer a prime example, we argue that it is necessary to consider computational reproducibility across different types of research assets. Drawing on existing frameworks that have driven critical changes to data sharing practices (e.g., Nosek et al., 2015), we aim to assess different forms of research assets as they relate to methods and tools published in *BRM*. Specifically, this builds on our previous paper (Towse et al., 2021a) in which we observed that, across a variety of psychology journals, most open data is neither complete nor reusable. In this regard, the work of Roche et al. (2015) in investigating the quality of data sharing in the field of ecology and evolution is critical. Rather than any formal attempt at computational reproducibility, they developed an ordinal scale of data quality based on principles. Likewise, Stodden et al. (2018) evaluated reproducibility on a qualitative scale (see their Table 4), from “Straightforward to reproduce with minimal effort” at one end to “Impossible to reproduce” at the other. One amusingly illustrative intermediate scale point was “Reproducible with substantial tedious effort”. In this respect, sacrificing a degree of quantitative precision offers some tangible benefits in affording ways to bridge different types of research activity.

Our goal is, therefore, to provide a higher-level overview of research assets, and our results will assist in understanding whether FAIR principles^3^ (on which our previous work on open data was founded) translate to other assets (Wilkinson et al., 2016). This will allow us to compare and contrast different research assets across the *BRM* corpus. The journal has recently implemented new policies to ensure that data and methods are open and encourage the depositing of code and worked examples. Papers are returned to authors on submission if no link to materials is provided (see Brysbaert et al., 2021). This policy was introduced for all new submissions on January 1, 2020 and subsequently made public as part of an editorial: “BRM requires the information to be easily available in a repository or in an appendix” (Brysbaert et al., 2021, p. 2). However, as has been seen with data sharing, mandates in themselves may not be enough to shift practices, and data sharing thresholds are often not sufficient or useful when *methods* must also be shared (Hardwicke et al., 2018; Hardwicke et al., 2020; Stodden et al., 2018; Towse et al., 2021a). By way of partial mitigation, *BRM*’s new policy operates at the point of submission and not further downstream after a paper has been accepted or published.

To explore how *BRM* policy is operating in practice, our preregistration posed the following broad research questions: (1) How quickly can methods and analytical techniques reported in *BRM* be used and developed further by other scientists; (2) Has functionality improved following changes to journal policy in support of computational reproducibility; (3) Can we disentangle such policy changes from changes in reproducibility over time (e.g., Vines et al., 2014)? Specifically, have changes to policy slowed the process of natural decline and improved the life span of research assets? While this study is exploratory in nature, we do have some specific hypotheses to test.^4^ For example, we hypothesize that journal policy changes will have an impact on the time taken to get assets running, on their completeness, and on their reusability. We will also explore whether policy changes have affected the “life span” of these research products, or whether the ageing effects remain the same. A natural decline function is be expected (i.e., the older a research output is, the less usable its assets may be), but it should be possible to test whether a change in journal policy can mitigate the inevitable impact of time on computational reproducibility.

Documenting the computational reproducibility of the research assets associated with *BRM* articles over time also has a broader and forward-facing objective. Specifically, by thus describing the landscape of research products in psychology, we seek to acquire evidence for the development of practical recommendations for improvement. By showcasing experience-based trajectories, we aim to promote a discussion about reasonable expectations on the part of researchers and the journal. Rather than seeking to be definitive, we endeavor to provide generative opportunities for more fine-grained research work with specific digital assets. This will support both *BRM* and researchers today and in the future.

## Method

### Pilot sampling

As part of a Stage 1 preregistered submission, we sampled 5% of *BRM* papers between 2014 and 2022 (the latter including those in-press) to better understand the different types of articles that appear in the journal and how they might be assessed for sustainability. In that period, approximately 1400 papers have been published and we observed from our sample that articles covered the results of surveys/experiments (20%), statistical code (30%), materials (25%), or software/hardware (20%), with other papers (e.g., reviews or corrections) accounting for the remaining 5%.

### Sampling strategy

Related research that has considered computational reproducibility or the quality of data sharing has sampled between ~70 and several hundred papers (e.g., Roche et al., 2015; Stodden et al., 2018; Towse et al., 2021a). In line with these norms and based on our pilot sampling, we randomly sampled an equal number of papers from the years before (2019 and 2020;^5^*n* = 102) and after (2021 and 2022; *n* = 102) the implementation of policy changes in order to provide useful conclusions (*N* = 204). Each paper was categorized as containing either data (usually from a survey or experiment), statistical code, materials, or software/hardware. Papers that reported on multiple assets were allocated a further, secondary category where applicable.

### Measures

After categorizing each research asset, we recorded whether the associated paper was available as an open-access publication (either gold or green). This is important, especially when, as acknowledged, a paper often serves as a ‘manual’ for understanding and utilizing the asset (e.g., materials, data etc.) that it describes; conversely, limited access to a paper can hinder individuals’ abilities to access and use its assets. We then recorded the number of minutes required to get a research asset open or running as expected (similar to Collberg & Proebsting, 2016; likewise, see Stodden et al., 2018). The ecological validity of this measure derives from the fact that most researchers will have a limited amount of time to get resources working before having to move on elsewhere. Thus, each researcher was afforded a 60-min time limit (per asset), which allowed ample time to verify that an asset was functional and to note any observations relating to accessibility or associated limitations. We also identified and coded for broken links, including those resulting from an institutional repository having removed materials.

Roche et al. (2015) developed an ordinal scale of data functionality for ecological and evolutionary research, which was then used in psychology (Towse et al., 2021a) and is therefore cited widely. We also know from some of the other work cited in our previous research that similar types of ordinal evaluation have been used in computational reproducibility (Stodden et al., 2018), among others. Thus, we scored *completeness* via an ordinal 1-to-5 scale, assessing what proportion of the resources and resource descriptors supporting a paper were publicly available (Roche et al., 2015; Towse et al., 2021a), with *reusability* also scored on an ordinal 1-to-5 scale, assessing how readily resources could be accessed and understood by third parties (Roche et al., 2015; Towse et al., 2021a). Accordingly, “5” is exemplary, “4” is good, “3” represents small omission/average, “2” represents large omission/poor while “1” is poor/very poor respectively. Either metric is commensurate enough for formal comparison between the two (i.e., to declare that the data is more functional – scores better – than the software or vice versa). While we expected these scores to correlate with the time required to access the identified resources/data or run the associated software, this may not always be the case. For example, software can score highly on completeness and reusability yet fail to run at the first attempt. However, a higher quality of resources is likely to mean that only small tweaks are required for them to become fully functional within our 60-min time limit.

### Analysis

#### Ensuring agreement between coders

We decided in advance that the entire sample would be double coded, with six pairs each coding 34 papers (204 in total). Raters first scored each asset independently. Having received this independent data from each coder, the lead author generated quantitative reports showing distributions of, and correlations between, key measures (time, completeness, and reusability) for each pair. Bland–Altman plots were also generated for each measure. This is a common method for analyzing and visualizing agreement between raters or methods of quantitative outcomes. In essence, a Bland–Altman plot is a scatter plot in which the differences between two measurements are plotted against their averages. This aids visualization of the degree of agreement between two raters and helps identify any systematic bias. Finally, an interclass correlation was reported for time and weighted kappas for completeness and reusability. An example report is available via the Open Science Framework (OSF) pages associated with this project (see Data/Code availability links).

Rather than serving as a formal analysis, these automated reports were used to help drive discussion when coding pairs then met to compare scores. This aimed to minimize variations in coders’ expertise and support pairs of coders in being exhaustive in their attempts to maximize computational reproducibility. In a similar fashion to Towse et al. (2021a), emergent issues were resolved by mutual discussion (e.g., the need for a coder to access a specific operating system, specific knowledge of a subject area, etc.). Following this second round of coding and discussion, each pair returned a final score for each of their papers to the lead author. No papers required checking by a third coder.

#### Final sample

**Fig. 1:**
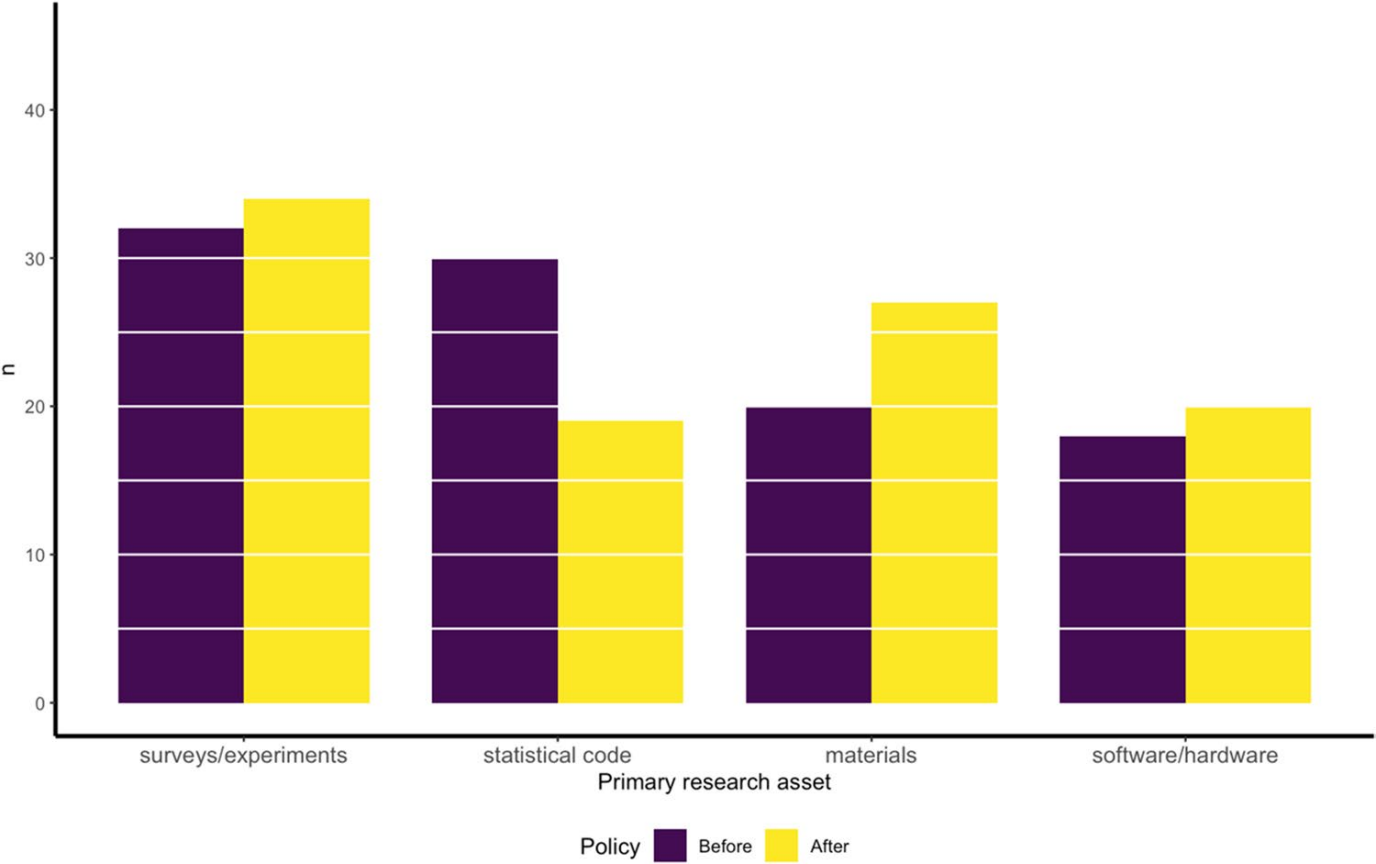
Counts by primary research asset showing the split between papers before and after journal policy changes; 61.5% of the sample was assigned a second category (for which the order of most to least common asset remained the same as shown here). A breakdown of which research assets were coded by each pair is reported in the Supplementary Materials (Table A)

A total of four papers were removed because they did not report on, or link to (e.g., via a correction or erratum), research assets relating to computational reproducibility. Specifically, one review, one erratum to a comment, one primer, and one commentary were not included in our final sample. This left a sample size of 200, a breakdown of which is presented in Fig. 1.

## Results

### How quickly can methods and analytical techniques reported in *BRM* be used and developed further by other scientists?

**Fig. 2:**
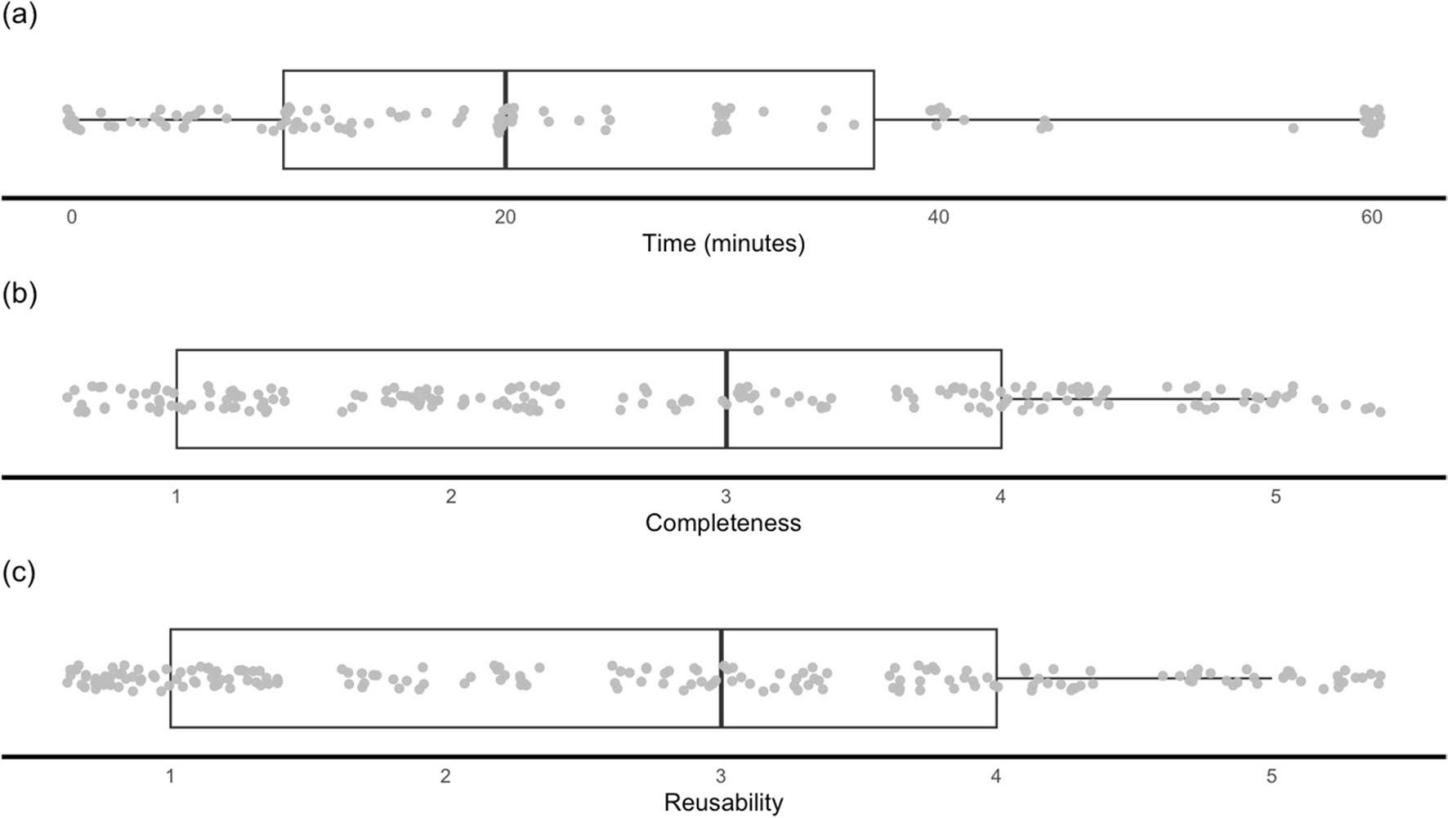
Distributions of time (**a**), completeness (**b**), and reusability (**c**) across the whole sample (*N* = 200)

On average, coders took 23.96 min (*SD* = 19.62) to access and use research assets. However, this time was usually only recorded when an attempt could be made to engage with assets in the very first instance (*n* = 120), otherwise time was coded as N/A. Scores for completeness (*M* = 2.76, *SD* = 1.41) and reusability (*M* = 2.68, *SD* = 1.48) were reported for all papers (see Fig. 2 for distributions of each metric across the sample as a whole).

### Has functionality improved following changes to journal policy in support of computational reproducibility?

**Fig. 3:**
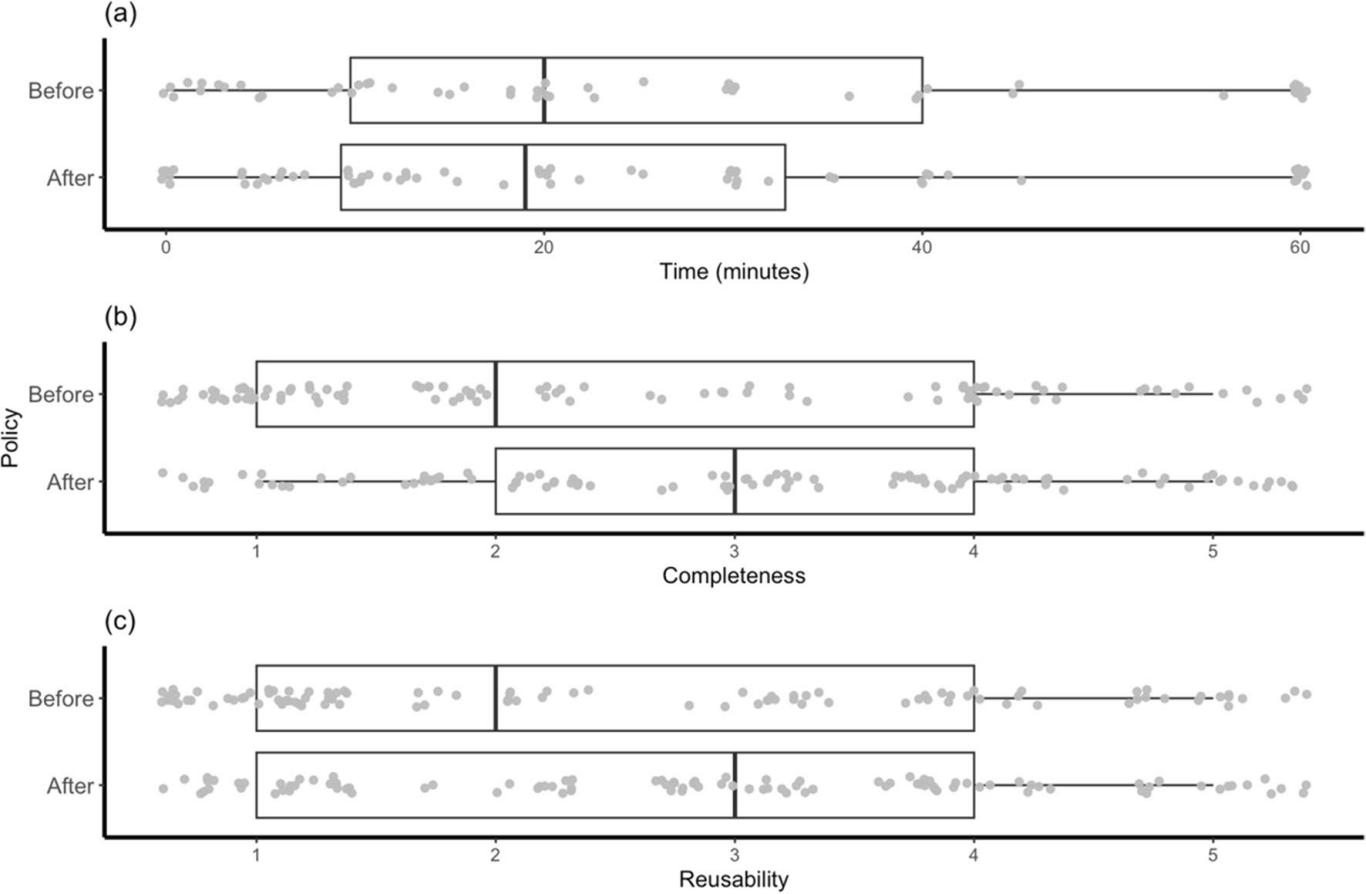
Distributions of time (**a**), completeness (**b**), and reusability (**c**) before and after changes to journal policy

Figure 3 shows the distributions of the same three measures when the sample is divided between articles published in the light of the current (i.e., changed) policy (current policy articles; CPAs) and those from an equivalent window beforehand (pre-policy articles; PPAs). While all measures show some improvement following the journal’s policy changes, it was only significant for completeness [*t*(198) = 2.64, *p* < .01; *d* = .37]. The changes in time [*t*(118) = – .79, *p* = .43; *d* = – .15] and reusability [*t*(198) = 1.87, *p* = .06; *d* = .27] between CPAs and PPAs were not significant (see Tables 1 and 2 for means and standard deviations).

**Table 1:** Means, standard deviations, Pearson and Spearman correlations (in bold) between time, completeness, reusability, and days since publication for pre-policy articles (PPAs)

	*M*	*SD*	1	2	3	4
1. Time (Before)	25.58	20.52		**.23**	**.03**	**.14**
2. Completeness (Before)	2.50	1.45	.20		.82^**^	**– .16**
3. Reusability (Before)	2.48	1.53	– .01	.80^**^		**– .18**
4. Days since publication (PPAs)	1683.45	230.16	.16	– .21^*^	– .20^*^	

Note: The correlations between time (Before) and other variables are based on *n* = 52

^*^Correlation is significant at a .05 level (two-tailed) ^**^Correlation is significant at a .01 level (two-tailed)

**Table 2:** Means, standard deviations, Pearson and Spearman correlations (in bold) between time, completeness, reusability, and days since publication for current policy articles (CPAs)

	*M*	*SD*	1	2	3	4
1. Time (After)	22.72	18.96		**.07**	**– .18**	**– .13**
2. Completeness (After)	3.02	1.33	.07		.74^**^	**.00**
3. Reusability (After)	2.87	1.41	– .17	.74^**^		**.11**
4. Days since publication (CPAs)	803.99	286.15	– .10	– .01	.11	

Note: The correlations between time (After) and other variables are based on *n* = 68

^*^Correlation is significant at a .05 level (two-tailed) ^**^Correlation is significant at a .01 level (two-tailed)

**Fig. 4:**
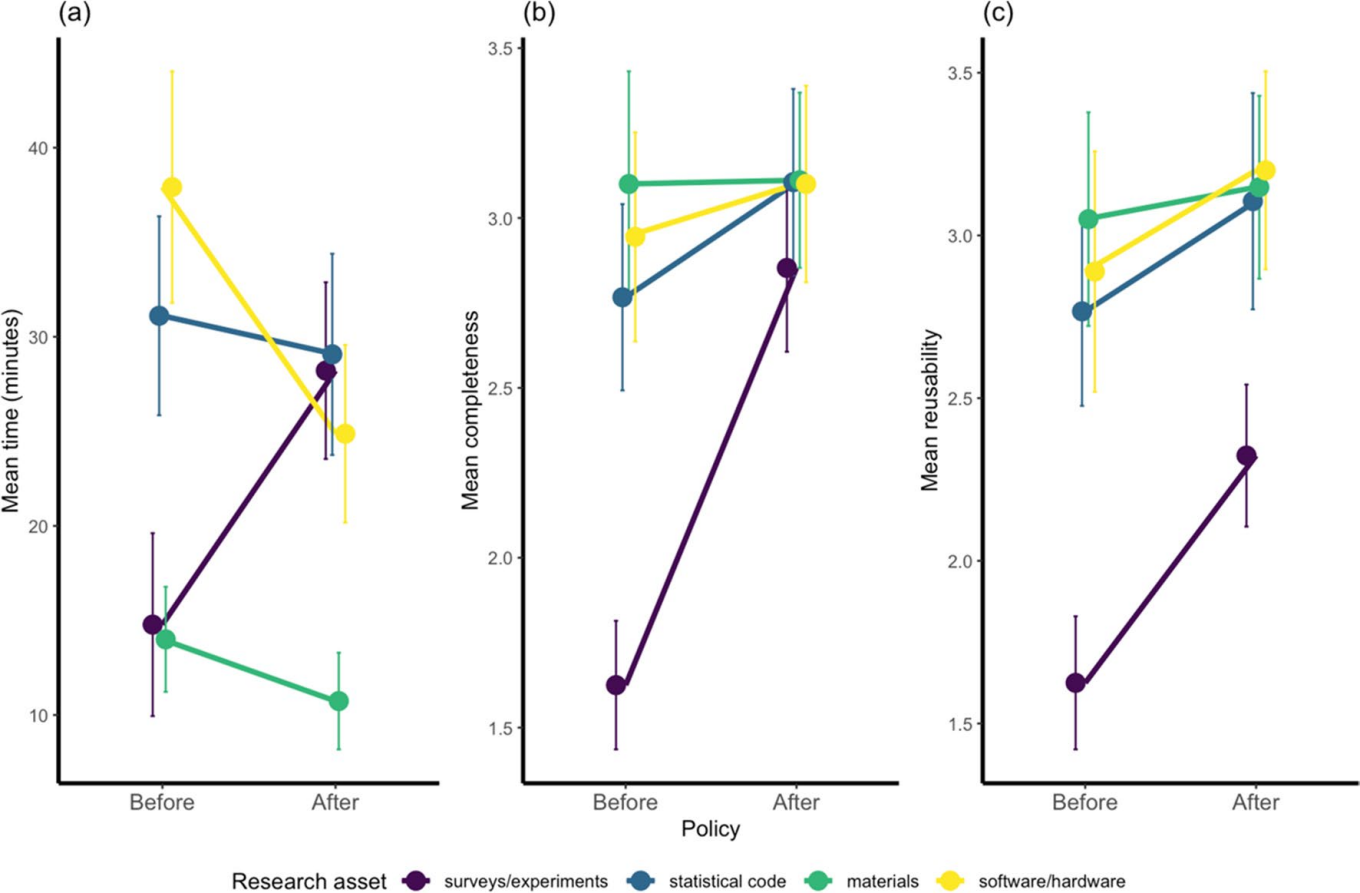
Average time (**a**), completeness (**b**), and reusability (**c**) among primary research assets before and after changes to journal policy. *Error bars* illustrate standard error. A breakdown of how assets were coded by each pair is reported in the Supplementary Materials (Fig. A)

While these improvements are encouraging, they were not evenly distributed across all categories of research asset (see Fig. 4). We computed a series of 2 × 4 ANOVAs to check for interactions between policy changes (before/after) and primary asset type (surveys/experiments, statistical code, materials, and software/hardware): no significant interactions were observed for time [*F*(3,112) = 2.36, *p* = .08, *η*^*2*^ = .05], completeness [*F*(3,192) = 2.39, *p* = .07, *η*^*2*^ = .03], or reusability [*F*(3,192) = .44, *p* = .72, *η*^*2*^ = .01].

We observed a main effect of policy in relation to completeness [*F*(1,192) = 4.94, *p* = .03, *η*^*2*^ = .02], rather than time [*F*(1,112) = .13, *p* = .72, *η*^*2*^ = .00] or reusability [*F*(1,192) = 3.12, *p* = .08, *η*^*2*^ = .01]. The main driver of this is likely to be improvements in the completeness of data from surveys/experiments, with far smaller changes to all other assets. It should also be noted that while not significant, the time taken to use the data from surveys/experiments was longer following the changes to journal policy. There were main effects of asset type on all three measures: time [*F*(3,112) = 7.16, *p* < .001,* η*^*2*^ = .15], completeness [*F*(3,192) = 5.10, *p* < .01, *η*^*2*^ = .07], and reusability [*F*(3,192) = 8.26, *p* < .001, *η*^*2*^ = .11]. Specifically, and irrespective of policy changes, the time taken to access materials was shorter, while the data from surveys/experiments was the least complete and reusable.

While the descriptive pattern is clear (see Fig. 4), the statistical outputs from the ANOVAs should be interpreted cautiously given the sample size and the between-subjects nature of the data. For this reason, we have not reported follow-up comparisons, but these can be computed from raw data located in the OSF.

### Can we disentangle such policy changes from changed reproducibility over time?

**Fig. 5:**
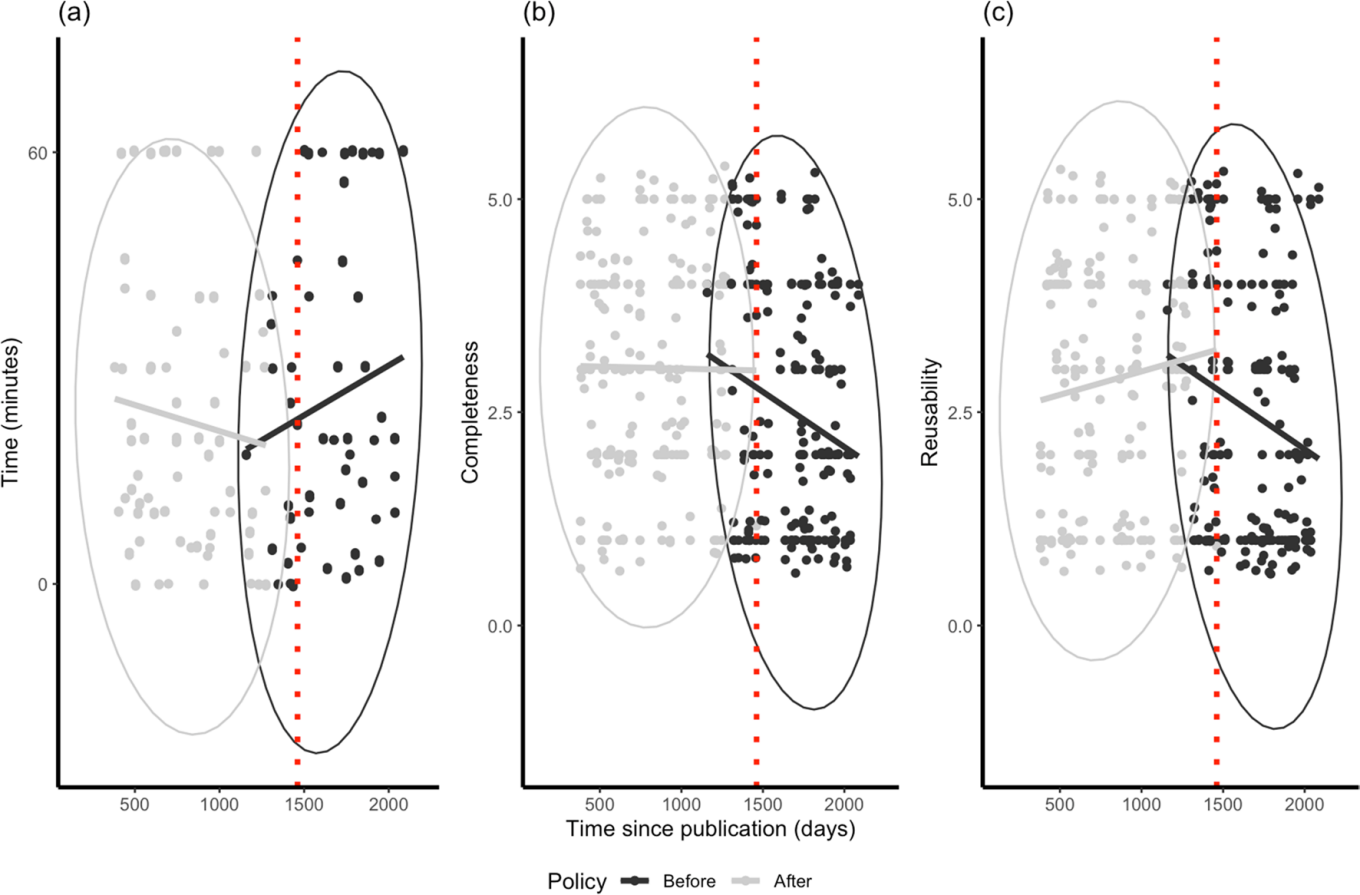
Measures of time (**a**), completeness (**b**), and reusability (**c**) plotted against time since publication for pre-policy articles (‘Before’) and current policy articles (‘After’). While higher values for completeness and reusability are proxies for improved quality, the direction of decline is reversed for time, where lower values are preferable (meaning assets were quicker to access and use). The *red dotted line* indicates where the policy change occurred: the majority of current policy articles (to the left) would have been submitted after January 1, 2020. See Fig. B in the Supplementary Materials for comparisons between research assets

The utility of all research assets may be degraded by the extended passage of time rather than as a result of any specific change in policy. In other words, being older, PPAs may be inherently less useful owing to natural ageing. We computed the time since publication for every article until January 1, 2024. Figure 5 plots decline functions for time, completeness, and reusability in relation to both PPAs and CPAs.

For PPAs, we can observe a consistent decline function: assets took longer to use and received lower scores for completeness and reusability as the number of days from publication increased (see Table 1). However, for articles published after the policy changes (CPAs), the decline is close to zero or even reversed (see Table 2).

Of course, it is possible that these CPAs will, over time, end up with an identical level of decay. By definition, the CPAs involve, on average, a significantly shorter time [*t*(198) = – 23.95, *p* < .001; *d* = – 3.39] since publication than PPAs (see Tables 1 and 2 for means and standard deviations). However, if regression lines were to be extended, these results suggest that any subsequent decline for CPAs will be less severe than that of PPAs, especially when one considers that the line for CPA completeness in Fig. 5 is flat, and those for time and reusability have reversed direction relative to their PPA equivalents.

## Discussion

In this preregistered report, we have explored how *BRM’*s authors and *BRM* policies can shape the landscape of functional research assets to support computational reproducibility. Looking across assets, scores of completeness and reusability are comparable to previous work that considered the quality of open data sets in a variety of psychology journals (Towse et al., 2021a). Similar challenges to those described therein were faced by our coders as they sought to access a variety of research assets. This included datasets that were difficult to understand, statistical code that was unclear, complex materials that were not clearly labeled, and software that did not run. While most papers were open access (green or gold), which in theory allows for the paper itself to act as a ‘manual’ for research assets, this rarely proved sufficient on its own. As a comparison, the best research assets provided a core point of access via a third-party repository (e.g., GitHub), had comprehensive documentation, offered a point of entry that demanded relatively low technical skills, and were regularly maintained or provided workarounds to avoid any natural degradation of essential dependencies.

We also sought to understand how changes to journal policy are changing the usefulness of research assets over time. *BRM* has implemented new policies that mandate open data and methods (see Brysbaert et al., 2021). This policy change was introduced for all new submissions on January 1, 2020. In this context, we observed one significant improvement, namely in the completeness of research assets. In other words, there were improvements to the publicly available resources and resource descriptors supporting a paper (Roche et al., 2015; Towse et al., 2021a). Reusability – assessing how readily resources can be accessed and understood by third parties (Roche et al., 2015; Towse et al., 2021a) – also improved and the time taken to access resources was, on average, reduced following changes to journal policy. However, this did vary by asset type. Statistical code, materials, and software/hardware were better regarding completeness and reusability before changes to journal policy with modest post-policy improvements. In contrast, data from surveys/experiments showed the most improvement in completeness and reusability. An associated increase in the time taken to access these data is likely to be due to greater quantities of data being accessible. This may well reflect the increasing attention being paid to the sharing of data across the psychological literature, and chimes with further guidance provided by the many journals that now mandate the sharing of data (Bauer, 2022; Brysbaert et al., 2021; Grahe, 2021; Sloman, 2015; Towse et al., 2021b).

A final analysis aimed to disentangle such policy changes from changes in computational reproducibility over time. The consistent associations so revealed suggested that journal policy changes are helping to protect against the inevitable impact of time. The decay of research assets of papers published after the changes to journal policy was less severe than that of assets in articles published prior to the changes. Decay trends are broadly similar between asset type although the strength of those correlations does vary (see the Supplementary Materials). However, the limitations of a reduced sample size (especially in relation to time) suggest the need for a cautious interpretation in this regard.

We are not aware of any other research that has examined the impacts on computational reproducibility across multiple assets before and after changes to a journal’s policy. *BRM* provides a somewhat unique opportunity in this regard. Perhaps the best direct comparisons can be made with Hardwicke et al. (2018) and Laurinavichyute et al. (2022), who considered data availability and analytic reproducibility before and after changes to open data policies at *Cognition* and the *Journal of Memory and Language*. Both studies observed improvements, but a finding common to these and Hardwicke et al. (2021), Crüwell et al. (2023), and Homewood (2023), who assessed reproducibility after changes to policies at *Psychological Science*, was that few papers, irrespective of changes to policy, supported full computational reproducibility. If full computational reproducibility equates to research assets scoring a 5 for completeness and reusability, only 16 (8%) of the papers in our sample met this criterion.

While our results are comparable in this respect, we, unlike Hardwicke et al. (2018) and (2021), did not contact authors for assistance, instead working with assets as they were presented. Our results may have been more favorable had we involved the original authors or brought in additional expertise. A focus on resolving ambiguity is important (as per the approach of Hardwicke et al.), but so too is our focus on longevity, because the opportunity for author assistance also declines over time.

## Limitations and reflections

Before making some recommendations and describing how these might shape the development of new policy, some limitations of our research are worth outlining in more detail. Perhaps more importantly, several of the associated reflections following our preregistered analysis raise broader issues that should be considered when planning future research.

Given publication lags, it is difficult to completely ensure that all of the papers treated as CPAs were actually submitted after the policy changes occurred. For example, a 2021-published paper could have been accepted before the policy changes were enacted. This information was not available to us, but our decline analysis, which involved recording the number of days since publication for each paper, reveals minimal overlap. However, it is important to note that this decline analysis was correlational and would require a longer-term follow-up before stronger claims regarding cause and effect could be made. It remains possible that articles published after the policy changes will, over time, end up with an identical level of decay to that of earlier articles.

While not all significant, each measure showed some improvement following changes to journal policy. However, these may have improved as a result of coders becoming better at accessing resources as they worked through their sample (practice effects). Papers were selected at random, and coders could work through them in any order, but lists were delivered to them such that the PPAs appeared before the CPAs. This fact was not intrinsically obvious to coders because the list simply contained DOI numbers and columns in which to record scores and notes. In further mitigation, Tables 1 and 2 reveal that associations between our three key measures were consistent. This suggests that the changes observed are unlikely to be the result of systematic variances in how CPAs and PPAs were assessed. Further discussion after initial coding is also likely to have limited any bias. We note that primary asset type was not balanced between pairs and while the split was broadly consistent with that represented in Fig. 1, some pairs, for example, assessed more statistical code or materials than data from surveys/experiments (See Table A in the Supplementary Materials for a full breakdown by pair). While unlikely, especially given an *N* of 34 per pair and a large variability between assets, it is difficult to determine categorically whether any of this variation has impacted our results.

In terms of specific measures, the number of minutes needed to access and get research assets up and running was less useful than anticipated. Sometimes it was simply not possible to access any assets and so the clock never started (N/A was recorded). Similarly, N/A was also recorded when assets appeared on first inspection to be available, only for coders to immediately hit a brick wall thereafter. Where a time was recorded, it could naturally be very short if assets were complete and reusable. However, more complex and well-documented assets that were equally complete and reusable could still take considerable time to set up. These practicalities likely explain why we observed no significant associations between time to access research assets, and completeness or reusability. Such a measure may indeed be more useful when comparing assets across a very narrow area of research (Collberg and Proebsting, 2016) or where resources have been provided after requesting materials directly from authors (Stodden et al., 2018).

Our focus was on quantitative research assets and, based on previous research, our measures were also quantitative in nature (Roche et al., 2015; Towse et al., 2021a). This perhaps disguises a somewhat more complex landscape. Put simply, even with a highly experienced team, accessing a variety of research assets was far from straightforward, which is difficult to completely capture with three quantitative measures. The exact difficulties faced by researchers were impossible to anticipate even with a preregistered design. Some of us succeeded or struggled simply due to (in)compatibility with our own platform of choice (e.g., Microsoft Windows vs. macOS, Chrome vs. Firefox), although some assets were designed for more than one platform. On several occasions an individual coder would leave a blank entry and wait on subsequent discussion to double-check their own technical expertise, always assuming that resources were easy to locate in the first instance (many were not). While coders aimed to be exhaustive in the efforts to locate resources, it is also possible that some assets were available but missed; for example, on an author’s personal website. That said, coders consistently managed to reach agreement after a follow-up discussion, with no papers requiring a third coder.

The foregoing discussion also raises the question of who the target audience might be for a specific asset. Our approach involved a team of researchers with varying expertise; as a team, we argue that we reflect the diversity of the wider research base. However, this creates something of a conundrum. If we were experts in the fields of work for all papers, and if we were experienced in handling all manner of compilers, interpreters, and their troubleshooting and debugging, then the speed and overall success of the checks might have improved. And yet, if that meant we only assessed assets that had been checked by deeply knowledgeable others (where we were, in effect, insiders for the work), this would surely produce a biased estimate of useability.

In contrast to subject matter that was not always quantitatively clear, all coders left copious notes detailing their experiences throughout the process. Some of those notes have helped inform our recommendations but a future thematic analysis (Braun and Clarke, 2006) might also be of benefit. For example, qualitative probe-based methods can uncover the cognitive processes involved when attempting to access and reuse assets as part of future research (Crandall et al., 2006; Eccles and Arsal, 2017). Think-aloud protocols specifically involve participants talking about their thoughts and experiences while moving around an environment (Evans and Jones, 2011), which here would likely involve opening files, attempting to get code running, and so on while simultaneously capturing out-loud thoughts and screen activity. The combination of qualitative and quantitative research could provide a deeper understanding of how various research assets are reused in practice and identify barriers to the further improvement of computational reproducibility. Such developments would also act as an additional means by which to engage the qualitative research community and its associated assets, which have different requirements when it comes to reuse and whose diversity of practice is unlikely to be addressed by current universal policy mandates (Prosser et al., 2024).

### Five recommendations (and their purposes)

Our results and reflections have allowed us to develop a series of recommendations. These deliberately avoid placing the responsibility on a single individual, team, or organization. Instead, our recommendations consider how researchers, journals, disciplines, and communities can work collectively to support computational reproducibility.

- *Researchers should provide clear and realistic resources to support research assets and computational reproducibility.* Creating clear documentation or tutorial materials involves, first of all, a realistic assessment of the requirements for use of specific research assets on the basis of their target audience. Questions to consider might include: How would a novice be guided to utilize this work? How might a collaborator or expert be guided instead? Could one set of documentation cater to both groups? For example, some statistical code may involve complex extensions to existing models that would only be of interest to those with some level of existing expertise in that area. In such cases, supporting documentation may not always need to be aimed at a novice and it would be fair to expect a researcher to gain the appropriate skills or seek expert guidance if there was a genuine interest in reusing such assets. Alternatively, where there is broad application and little specific expertise required, then the supporting materials should be developed to be followed by the majority. To support these efforts, journals should offer checklists and templates accordingly.
- *Journals should standardize how resources are identified and optimally managed.* Emphasizing our previous recommendations in relation to data sharing (Towse et al., 2021a), a core part of every paper should signpost resources and point to where these are available. The variability in how research assets and resources are identified within a paper have, to date, been driven largely by researchers themselves and their resources (including time and reward incentives). For journals, this means providing guidance on where and how to locate resources, which would also help with the automation of research asset identification. This includes standardizing the findability of assets once a reader leaves the immediate system (e.g., via a DOI). For example, third-party repositories such as GitHub can be linked to Zenodo to provide permanent URLs. If authors know exactly where in their article to describe their research assets, it will provide a tangible structural incentive and behavioral nudge. Likewise, if readers know where to look, the use of research assets will be simplified and encouraged.
- *Psychology as a discipline should ensure the provision of support for computational reproducibility.* Researchers, reviewers, and editors require resources and skills to curate research assets and supporting documentation in ways that align with computational reproducibility. The identification and collation of existing resources^6^ are already helping build capacity to support reproducible manuscripts (Hardwicke & Vazire, 2024), but journals, funders, and institutions could go further by providing additional support, especially given the breadth of research assets now being developed by psychologists. Developing resources to support research assets can sometimes require a skillset that the original researchers do not possess, and those who write code are not always best placed to write non-technical instructions. Going further, training and guidance should also include the consideration of where it may not be sensible to share materials or methods if they are in a format that might be weaponized or used to compromise an individual’s privacy or security (Dennis et al., 2019). All of the above needs to be integrated in a consistent fashion as part of the training for doctoral students. Beyond psychology, other disciplines may want to emphasize different ways of handling such changes.
- *Communities should prioritize and work toward appropriate standards for the longevity of research assets.* While it might be premature to stipulate an ideal period for the functional lifetime of a research asset, further consideration is required as authors and journals aim to implement long-lasting changes to policy. Authors should assume that their assets must be sustainable, and this involves the use of DOIs, and so on, to ensure longer-term archives. In the short term, one simple approach would be to develop a system for recording “when last tested,” giving a visitor some insight into whether an asset is likely to have become obsolete. This might leverage online systems to allow reports from researchers in which they flag whether or not resources are still working, and could be extended further to enable active discussion. Longer term, this would allow future projects to consider the challenges associated with longevity across a much larger sample of research assets and help determine what might be “normal” in terms of extended functionality within psychology. Of course, this not just an issue idiosyncratic to psychology or *BRM*. Cross-disciplinary efforts to understand and communicate how software and method artifacts can be made as useful as possible for as long as possible are continuing (e.g., Piccolo & Frampton, 2016; Katz & Chue Hong, 2024; Ziemann et al., 2023). Psychology can therefore benefit from excellent work being done in disparate places, that nevertheless addresses commonalities. That said, such developments also speak clearly to how increased longevity requires a concerted effort from the creators of resources in tandem with the development of associated infrastructures and support systems.
- *Funders and employers should recognize research asset curation and maintenance.* Many research assets require regular maintenance in order to remain useful. Sometimes this will involve simple changes to code, but in other cases (especially in relation to software) this may require sizable modifications to comply with changes to operating systems or data protection policies. New features may also be added during this time. When assets are freely available, such modifications often involve people who were not authors of the original publication. Therefore, providing tangible recognition for asset maintenance is important. Of course, republishing work with updates does open the possibility of gaming the system, for example, by publishing sham updates to increase the total number of research outputs. However, this could be mitigated by requiring a clear justification and explanation for each modification. The main point is that funders and institutions should consider how best to recognize and incentivize work in asset creation and maintenance that is very different, but no less important, than the publication of a paper that might otherwise be rendered increasingly less useful.

### Future policy and wider challenges to computational reproducibility

While the results from *BRM* suggest that matters there are heading in the correct direction for quantitative research, recent developments at other psychology journals have continued at pace. In response to some of our previous work (Towse et al., 2021a), and during the write-up of these results, *Psychological Science* has reformed its guidance (Hardwicke & Vazire, 2024). In line with the policies of many other journals (including *BRM*), it requires original research materials and analysis scripts to be made publicly available in a trusted third-party repository. If these are not present, it must be clearly justified in a Research Transparency Statement. Clear documentation that describes data or analysis scripts is also an essential requirement now. These changes are complemented by the decommissioning of Open Science Badges and the introduction of a new Computational Reproducibility Badge, awarded when authors take the necessary steps to ensure that reported results can be independently reproduced, within a reasonable time frame, by newly appointed Statistics, Transparency, and Rigor (STAR) editors who can provide specialized support and perform a variety of verification tasks.^7^ We view all of these developments as encouraging and very much in line with our own recommendations (see Hardwicke & Vazire, (2024 for full details). Many of these changes also mirror those of leading journals in other fields, including economics and medicine (Loder et al., 2024; Vilhuber, 2020).

In practice, these changes to policy point toward larger challenges in how academic articles are published and archived. For example, one paper (which appears in *BRM*) reports on open-source software (Geyer et al., 2019). It has only remained functional as a result of extensive revisions of the source code by a new author who is credited on a postprint (Geyer et al., 2020). While the third-party repository (in this case GitHub) has been updated, publication systems are not set up to support the addition of authors post-publication or the updating of broken links and so on, despite this all being technically possible. Authors can, of course, write computationally reproducible papers (see Lakens and DeBruine, 2021), but this relies on code to generate analysis and data visualizations in real time in support of computational reproducibility and at some point, once published as a PDF, these effectively become static. Authors are forced to rely on specific sections of a paper that point to third-party repositories, yet a more advanced solution would utilize automated workflow pathways to support research assets (e.g., González-Beltrán et al., 2015; Sharma et al., 2023). Code, for example, could run directly within an online publication, but still sit in a third-party repository that could be updated over time without requiring major changes to the final journal article. Building on systems that can automatically uncover datasets associated with research papers (e.g., https://dataseer.ai/), generative AI systems could go further by updating code based on published changes to programming libraries or other dependencies to improve and extend the lifespan of other research assets. In other words, publishers, as well as the journals themselves, have a role to play in supporting these endeavors today and in the future, especially as the value of what counts as an academic output continues to shift.^8^

We have analyzed the changes and developments taking place and discussed improvements for specific journals such as *BRM* and *Psychological Science*. Do these constitute ‘exceptions that prove the rule’ that little real innovation is taking place, or do they truly represent the vanguard of change? We observe increasing levels of polarization across and between journals, funders, and institutions. For example, while several journals lead and others follow, some appear to be falling behind and show little interest in the transparent reporting of results or research assets in support of computational reproducibility (see, for examples, Towse et al., 2021a). Journals can now be further compared following the introduction of TOP (Transparency and Openness Promotion) Factors.^9^ These metrics report the steps that a journal is taking to implement open research practices that also align with the support of computational reproducibility. Funders also vary in their expectations and how these are implemented. For example, UK Research and Innovation (UKRI) expects research papers from funded work to be open access and data arising from its funding to be made as open and unrestricted as possible.^10^ However, there has yet to be any research into the effectiveness of data sharing as part of the research funded by UKRI, or how other research assets are shared in a way that supports their reuse and computational reproducibility. This offers the potential for another interesting exploration of how well things work in practice for a variety of research assets. Similarly, institutions are inconsistent in how academics are supported and rewarded when engaging in such endeavors. Guidance is readily available for institutions and associated academic systems in support of associated change (e.g., Kohrs et al., 2023; Rahal et al., 2023; Weinerova et al., 2024), but without sizable cultural shifts, institutions will continue to “reinforce research practices that are known to be problematic while insufficiently supporting the conduct of better-quality research and open science” (Rice et al., 2020, p. 1).

## Conclusion

Here, we have shown that journal policy changes at *BRM* are having a positive impact. As with other core processes linked with research integrity (including ethical practices; Bauer, 2020), this will spur further discussion of what else might change in the future. How this plays out will be intrinsically linked to how the academic community and supporting research entities react and collectively curate policy. Therefore, our recommendations emphasize a coordinated approach that does not solely involve researchers themselves, but also requires changes to a complex ecosystem that will help the discipline move forward faster and progress further.

We also must acknowledge that the path forward should reflect the interdisciplinary landscape in which psychology finds itself and that improvements can be mutually beneficial for our own and cognate disciplines. This includes working with research support staff (e.g., librarians) as well as engaging in cross-disciplinary collaborations (Farrell, 2023). Psychology in particular, however, remains in “an intense period of reform, growth, and self-reflection” (Vazire, 2024, p. 1) and ensuring that all research assets are available *and* useful remains core to supporting researchers and research users in maximizing a collective good (Tiokhin et al., 2023). Changing journal policies to reflect that aim, while less than straightforward to assess, clearly remains a worthwhile endeavor.

## Supplementary Information

Below is the link to the electronic supplementary material.

Supplementary file1 (DOCX 608 KB)

## Data Availability

All data and materials, including our pre-registration plan, are available on the Open Science Framework (OSF) [https://osf.io/3ygua/wiki/home/].

## Footnotes

1 To improve this situation further, *Psychological Science* now requires that all original research materials, including data and analysis scripts, should be made publicly available in a trusted third-party repository (Vazire, 2024).

2 https://www.software.ac.uk/

3 FAIR principles focus on how data should be Findable (e.g., have a persistent identifier and descriptive meta-data), Accessible (ideally, available without authentication requirements or data sharing restrictions, with metadata to clarify any conditions or accessibility issues even if raw data are not available), Interoperable (use common standards for description), and Reusable (appropriately licensed and meaningful).

4 See this page for a complete list of hypotheses (10.17605/OSF.IO/HJC45).

5 As far as possible, we ensured that papers published in 2020 had been submitted before January 1, 2020.

6 See a primer series from the UK Reproducibility Network, which is designed to introduce a broad audience to important topics in open and reproducible scholarship (https://www.ukrn.org/primers/).

7 Related mechanisms have been developed at some institutions whereby researchers can have an independent statistician check and certify the computational reproducibility of papers before they are submitted for publication (e.g., https://www.ukrn.org/2024/01/30/pre-submission-certification-of-computational-reproducibility/).

8 https://www.ukri.org/news/early-decisions-made-for-ref-2028/

9 https://topfactor.org/

10 https://www.ukri.org/manage-your-award/publishing-your-research-findings/

## Code availability

Code to generate Figures is available on the Open Science Framework (OSF) [https://osf.io/3ygua/wiki/home/].
