# Supplementary material for: Assessing computational reproducibility in Behavior Research Methods
Source: Behav Res Methods. 2024 Sep 25;56(8):8745–60. doi: 10.3758/s13428-024-02501-5 (PMC11525395; doi:10.3758/s13428-024-02501-5)

| **Table A** Breakdown of assets coded by pair | | |  |  |  |  |  |  |
| --- | --- | --- | --- | --- | --- | --- | --- | --- |
|  |  |  |  |  |  |  |  |  |
| Primary research asset | Surveys/experiments | | Statistical code | | Materials | | Software/hardware | |
|  | *n* | % | *n* | % | *n* | % | *n* | % |
| Pair |  |  |  |  |  |  |  |  |
| One | 7 | 10.61 | 8 | 16.33 | 10 | 21.28 | 9 | 23.68 |
| Two | 10 | 15.15 | 12 | 24.49 | 6 | 12.77 | 6 | 15.79 |
| Three | 13 | 19.70 | 6 | 12.24 | 10 | 21.28 | 4 | 10.53 |
| Four | 14 | 21.21 | 10 | 20.41 | 3 | 6.38 | 7 | 18.42 |
| Five | 15 | 22.73 | 8 | 16.33 | 5 | 10.64 | 5 | 13.16 |
| Six | 7 | 10.61 | 5 | 10.20 | 13 | 27.66 | 7 | 18.42 |

**Fig. A** Average time (**a**), completeness (**b**), and reusability (**c**) split between research asset and coding pair. *Error bars* illustrate standard error


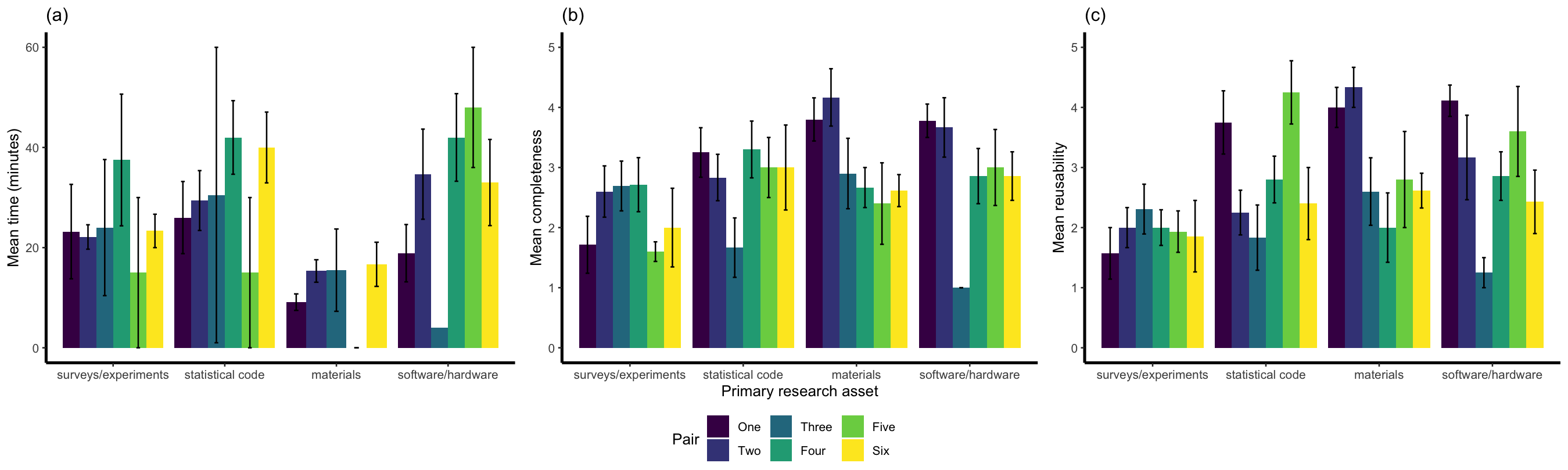


**Fig. B** Split by research asset: Measures of time (**a**), completeness (**b**), and reusability (**c**) plotted against time since publication for pre-policy articles (‘Before’) and current policy articles (‘After’). While higher values for completeness and reusability are proxies for improved quality, the direction of decline is reversed for time, where lower values are preferable (meaning assets were quicker to access and use). The *red dotted line* indicates where the policy change occurred. Pearson *r* values are also reported


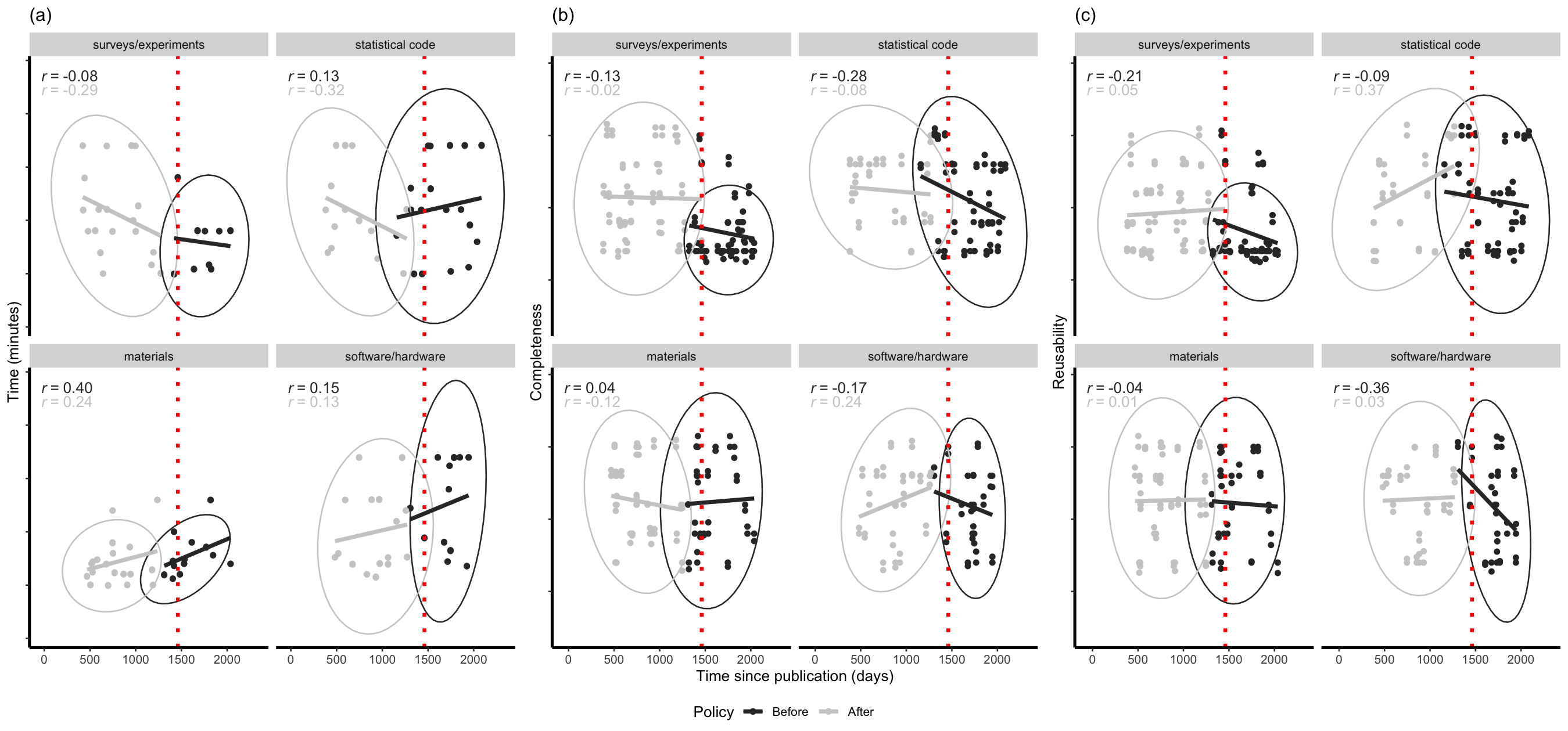

Supplement: Supplementary file 1 — Supplementary file1 (DOCX 608 KB) [file 13428_2024_2501_MOESM1_ESM.docx]
